# Supplementary material for: Plastome Evolution, Phylogenomics, and DNA Barcoding Investigation of Gastrochilus (Aeridinae, Orchidaceae), with a Focus on the Systematic Position of Haraella retrocalla
Source: Int J Mol Sci. 2024 Aug 4;25(15):8500. doi: 10.3390/ijms25158500 (PMC11312641; doi:10.3390/ijms25158500)
Supplement: Supplementary file 1 [file ijms-25-08500-s001.zip › Zhou et al. SI.pdf]

Supplementary materials

Table S1. Plastome characters of 16 *Gastrochilus* species.

Table S2. Summary of repeats sequences of 16 *Gastrochilus* plastomes.

Table S3. Morphological characters used for PCA in this study.

Table S4. Taxa, source and GenBank accession numbers used in this study.

Figure S1. Mauve alignment of the complete plastome of 16 *Gastrochilus* species. The color band with the same color in the figure is a local collinear block, representing a set of homologous genes. The lines linking the collinear blocks represent homology between different genomes. The two rows below the color band represent the genes. The upper side is on the positive chain, and the lower side is on the complementary chain. The white squares represent CDS, the thin lines in the white squares represent introns, and the green and red squares represent tRNA and rRNA respectively. Numbers on the upper x-axis are genome map coordinates in kilobases (Kb). The topology on the left was the ML tree based on plastomes with IRa excluded.

Figure S2. Heat map of codon usage bias in the plastomes of *Gastrochilus* under RSCU. Red and blue indicate higher and lower RSCU values, respectively.

Figure S3. Comparative analysis of sixteen *Gastrochilus* plastomes using the

mVISTA program with *Pomatocalpa spicatum* as a reference. Grey arrows above the alignments indicate gene orientations. Genome regions are color-coded as protein-coding (exon; purple), untranslated regions (UTR; cyan), and conserved non-coding sequences (CNS; pink). The x-axis represents the coordinate in the chloroplast genome. The vertical scale indicates the percentage of identity, ranging from 50 to 100%. The topology on the left was the ML tree based on plastomes (excluding IRa).

Figure S4. Principal component analyses of 46 *Gastrochilus* and *Pomatocalpa* species based on 14 morphological characters. Colored dots and regions stand for *Gastrochilus*, *Haraella* (*G. retrocallus*), and *Pomatocalpa*, respectively. On the right side, pictures demonstrate the vegetative and floral morphology of *G. calceolaris* (A, B); *H. retrocalla* (*G. retrocallus*; C, D); and *P. spicatum* (E, F). Pictures were taken by the authors (A, B, F) or downloaded from http://ppbc.iplant.cn/ (C, D, E).
