# Supplementary figures and images for: Plastome Evolution, Phylogenomics, and DNA Barcoding Investigation of Gastrochilus (Aeridinae, Orchidaceae), with a Focus on the Systematic Position of Haraella retrocalla
Source: Int J Mol Sci. 2024 Aug 4;25(15):8500. doi: 10.3390/ijms25158500 (PMC11312641; doi:10.3390/ijms25158500)

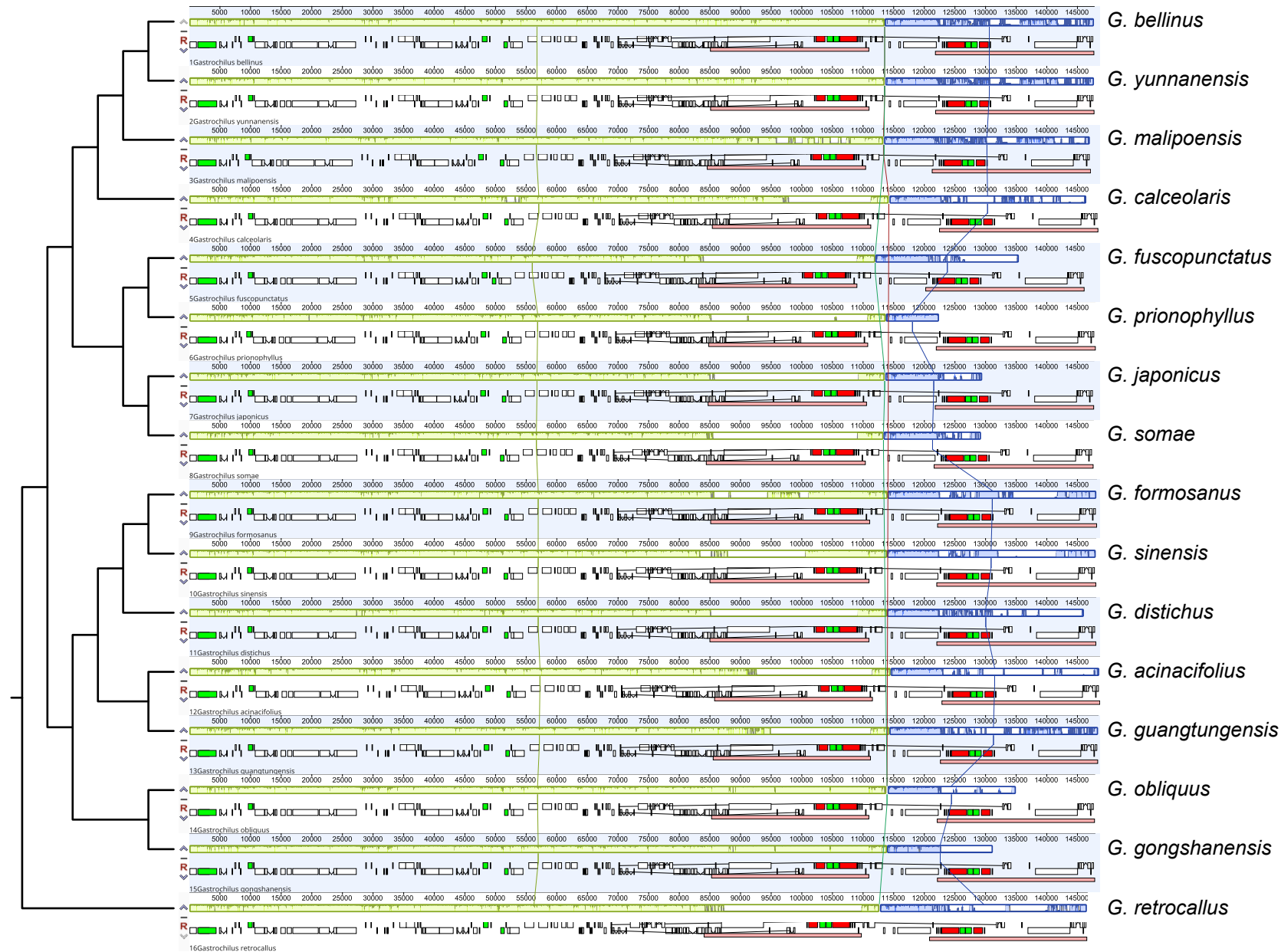



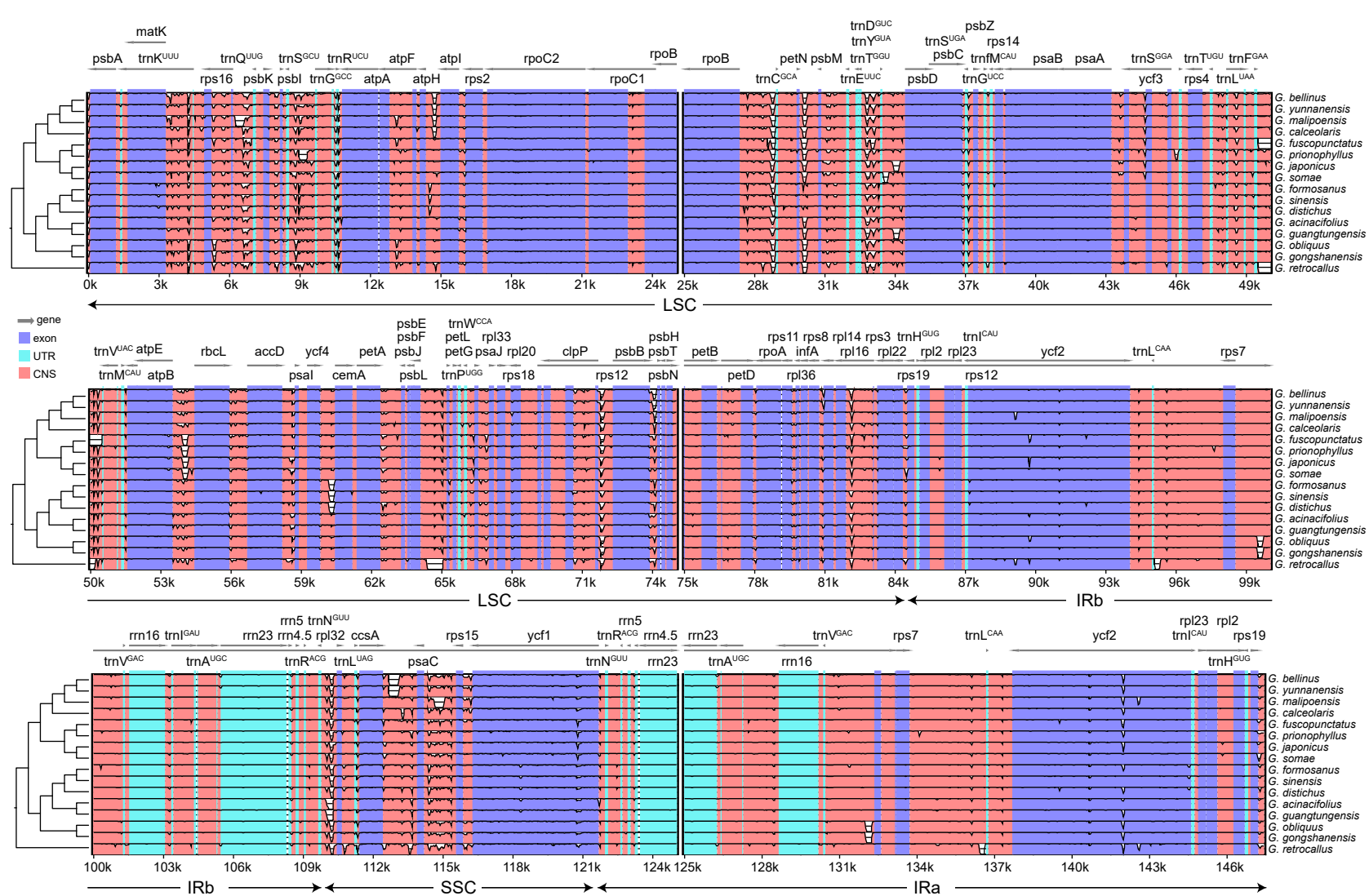

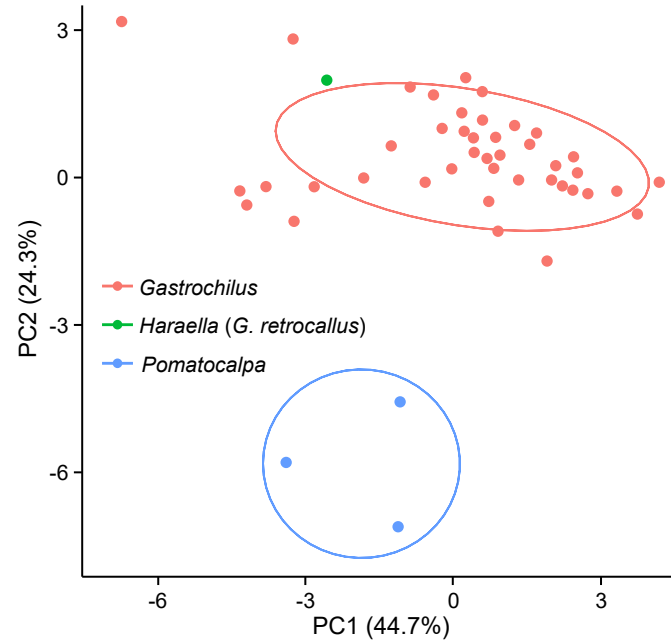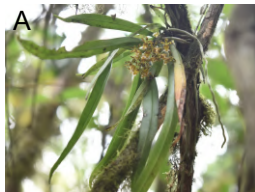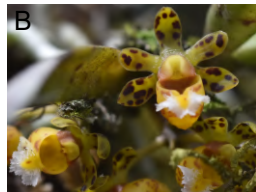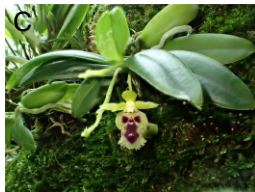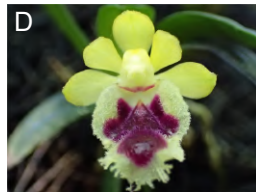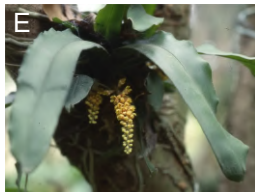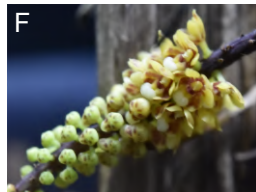

Supplement: Supplementary file 1 [file ijms-25-08500-s001.zip › Figures S1-S4.pdf]
